# Balancing Urgency and Care: Discursive Perspectives on Anxiety‐Related School Refusal in Adolescents—Institutional, Nursing, and Transcultural Insights From a French Maison des Adolescents

**DOI:** 10.1002/nop2.71028

**Published:** 2026-11-10

**Authors:** Rahmeth Radjack, Alice Titia Rizzi, Nelly Massari, Delphine Brown, Aurélie Harf, Eglantine Mazeaud, Christine Baveux, Marie Rose Moro

**Affiliations:** ^1^ Maison de Solenn, Cochin Hospital Paris France; ^2^ Université Paris Cité, Laboratoire de Psychologie Clinique, Psychopathologie, Psychanalyse (PCPP) Boulogne‐Billancourt France; ^3^ CESP, UVSQ, Inserm, Team DevPsy Villejuif France

**Keywords:** adolescence, anxiety‐related school refusal, multidisciplinary care, multifamily therapy, nursing, transcultural psychiatry

## Abstract

**Background:**

Anxiety‐related school refusal is a complex phenomenon that raises questions at the intersection of psychiatry, education, and family systems.

**Aims:**

This perspective article examines therapeutic and institutional approaches developed in a French *Maison des Adolescents*, a specialised multidisciplinary centre for adolescent mental health. We discuss the tensions between urgency expressed by families and schools, and the need for a graded reintegration whose timing is set by the adolescent's clinical condition.

**Materials and Methods:**

Clinical vignettes illustrate the overlap between school refusal, comorbid psychiatric symptoms, and sociocultural factors, including migration and trauma.

**Results:**

We argue that care must be conceived on three interconnected levels: individual, family, and environmental. Options such as weekly day hospital programmes, multifamily therapy, and transcultural consultations demonstrate both strengths and limitations.

**Discussion:**

For and against arguments are highlighted: while institutional programmes provide containment and support, they may also risk premature or excessive intervention.

**Conclusion:**

The central question addressed is: How can multidisciplinary adolescent care centres best support young people with anxiety‐related school refusal while balancing urgency, family dynamics, and educational reintegration?

**Implications for Nursing Practice:**

First‐contact nursing assessment combines relational engagement, physical and mental‐health assessment, and risk identification to guide timely referral and an agreed care plan. Nurses engage adolescents and families, help contain pressure for immediate school return, and contribute to care coordination and collaborative, graded school reintegration adjusted to the adolescent's clinical condition. Ultimately, anxiety‐related school refusal must be approached as a systemic issue requiring joint solutions from health, education, and family contexts. Our reflections aim to inform international practice and stimulate debate on therapeutic, institutional, and transcultural options for managing this condition.

## 1 Introduction

Anxiety‐related school refusal is encountered in mainstream schools, in child psychiatric services, and in the private practice of child psychiatrists, paediatricians and family doctors. In France it is commonly called school phobia. Its growing visibility should not be equated with a demonstrated rise in prevalence. It is met within an educational context in which schooling is compulsory until 16 and in which families attach considerable importance to the baccalauréat, often seen as a rite of passage into adulthood and as the guarantee of the widest possible choice before committing to a specialised or vocational pathway.

This academic framing is not the only one adolescents have available. Many invest in domains—sport, music, digital creation, online audiences—in which competence is recognised quickly, by peers, and on criteria that are not the school's. What counts as ability is itself culturally coded (Mazeaud et al. 2023), and the distance between what a family expects and what an adolescent finds recognised elsewhere may sharpen the sense of failure that attendance comes to carry. In transcultural situations the stake can run deeper still: school engagement may itself be experienced as a threat of rupture with familial and cultural roots, a configuration described elsewhere as a roots breach (Rizzi et al. 2026).

Anxiety‐related school refusal has historically been confused with truancy or with academic failure, although its identification varies across sociocultural contexts (Brault et al. 2022). The term school phobia is both widespread and contested, since the difficulty does not correspond to a specific phobia but to the anxiety that comes to attach to the school as an object. Medical nosography increasingly favours anxiety‐related school refusal, which names the mechanism at work: the young person wishes to attend and finds themselves unable to.

Terminology in this field is not settled, and the distinctions matter clinically. Following Heyne et al. (2019), we use *school attendance problems* as the umbrella term covering all forms of non‐attendance; *truancy* for absence concealed from parents and not driven by emotional distress; *school refusal* for absence of which parents are aware and in which the young person is unable to attend despite wishing to; and *anxiety‐related school refusal* for the subset in which anxiety is the principal mechanism. *School phobia*, still current in French clinical and lay usage, is retained here only when reporting how families, schools and practitioners name the problem, since the phenomenon does not correspond to a specific phobia. *Absenteeism* is used descriptively, as a measure of missed schooling, and not as a diagnostic category. The clinical discussion that follows concerns anxiety‐related school refusal; where the studies cited use broader or different definitions, this is indicated.

Anxiety‐related school refusal has particular features during adolescence. Early signs may already have been present in childhood without leading to complete withdrawal, but referral for established school refusal is more common among adolescents, and the first 2 years of secondary school appear to be a peak period for referral. That transition coincides with the changes of puberty and with entry into a larger and more complex school environment: a wider peer group, multiple teachers, greater academic demands, increased expectations of autonomy. These changes may expose or intensify pre‐existing vulnerabilities, and established school refusal in adolescence is often more severe and complex than the difficulties seen earlier in childhood (Heyne et al. 2014; Ingul et al. 2019).

International studies report prevalence rates and describe the psychopathological disorders associated with school refusal, but few describe the therapeutic pathway in any detail. School refusal generates considerable stress for parents and for professionals unfamiliar with it, who therefore look for immediate solutions; that urgency is often mirrored in hasty decisions such as requests for hospitalisation or rapid re‐enrolment in distance learning.

Here we present and examine the model of care developed at our Maison des Adolescents for adolescents with anxiety‐related school refusal, with particular attention to the nursing role at first contact and to the risks of interventions whose timing or intensity does not fit the clinical situation. The central question of this paper is: how can multidisciplinary adolescent care centres support young people with anxiety‐related school refusal while balancing the need for timely intervention with the risks of premature or inappropriate decisions?

## 2 The *Maison des Adolescents*

Adolescent centres (maisons des adolescents) are open to the general adolescent population. Created in France in 2008 with the aim of establishing one per department, they emphasise welcoming adolescents and reducing the stigma attached to mental health care (Benoit et al. 2018). There are now 126 such centres, including in the overseas territories (Association Nationale des Maisons des Adolescents 2026).

Our centre receives young people with a wide range of difficulties related to adolescence. It is at once preventive and curative, works in close connection with local care networks, and acts as a bridge between the outside world, the health care system and family life.

Nurses have a specific first‐line role in this structure. They are often the first professionals to meet adolescents and their families, either by telephone or on site without a prior appointment. Their intervention consists of a brief, relational and clinically oriented assessment aimed at identifying needs, facilitating engagement with care, and guiding the adolescent and family towards the most appropriate form of support. Nurses usually meet first with the adolescent and the parents together and then with the adolescent alone. Because the initial request often comes from the parents, particular attention is paid to eliciting the adolescent's own understanding of the situation and, when necessary, helping an initially unformulated request for care to emerge. Nurses begin with the adolescent as a person, including their abilities, interests and wishes, and introduce more sensitive questions progressively so that the interview is not experienced as intrusive.

Suicide‐risk assessment is conducted sensitively, with relationship‐building informing how questions are approached rather than delaying their assessment. When self‐harm, suicidal ideation, acute deterioration, or other risk indicators are present, an immediate safety assessment is undertaken. Any reported suicidal thoughts are explored further. The nurse then determines whether the adolescent should first be reviewed by a general practitioner, referred to community, private or voluntary‐sector care, or seen by a physician within the Maison des Adolescents who will coordinate further treatment. At the end of the assessment, the adolescent and parents are brought together, the proposed care plan is explained clearly, and their agreement is sought. A safety plan may also be established when suicidal thoughts are present. The immediate nursing objectives are therefore the emergence of the adolescent's own account, identification of physical and psychological needs and risks, engagement of the adolescent and family in an agreed care plan, and appropriate orientation within or outside the institution. These are not outcomes we have measured. Within the multidisciplinary team, nurses also contribute to care coordination and continuity, linking adolescents, families and other professionals. These roles are developed further in the Implications for Nursing Practice section.

## 3 Clinical and Institutional Approaches

### 3.1 A Relevant Multidisciplinary Approach to Diagnostic Uncertainty

Anxiety‐related school refusal is one of the main reasons for consultation at the Maison des Adolescents, where it represents approximately one quarter of our current caseload. The figure comes from our own service activity; we know of no reliable national estimate for France. Multidisciplinary management, combining psychiatric and paediatric perspectives, is well suited to situations of diagnostic uncertainty.

In some situations the presentation is clearly psychiatric. In others, the route to a diagnosis is obscured by somatic symptoms of anxiety: stomach pain on school days, marked fatigue that may reflect unconscious avoidance, recurrent discomfort preventing regular attendance, or somatoform presentations leading to extensive gastroenterological or internal medicine investigation. Such presentations are well documented, physical complaints often masking psychiatric distress (Li et al. 2021; Ludot‐Grégoire et al. 2022). In our experience the underlying difficulty is sometimes identified only after somatic investigation has been pursued at length.

More broadly, any severe psychological suffering during adolescence—regardless of its nature—can affect schooling. It is therefore crucial not to overdiagnose anxiety‐related school refusal, as its management differs from that of depressive, post‐traumatic, anxiety, psychotic, or autism spectrum disorders.

Behind the term school phobia lie multiple psychopathological configurations. Clinical experience at our centre suggests two orders of question: what the young person may fear, and the situations in which an anxiety‐related meaning is at risk of going unrecognised, often in ways that are only partially conscious (see Table 1). Separation anxiety and concerns about parental vulnerability are recurrent themes in our consultations and have been described in the French clinical literature (Fejtö 2024). Experiences of bullying, past or ongoing, have been associated with school refusal and with anxiety symptoms (Egger et al. 2003).

**TABLE 1:** Psychopathological meanings underlying anxiety‐related school refusal. The two columns answer two different questions and are not mutually exclusive. The first sets out meanings that the young person may attribute, explicitly or not, to going to school. The second sets out situations in which an anxiety‐related meaning is at risk of going unrecognised, whether by the family, the school, or the clinician. Each entry is a hypothesis to be explored with the young person and the family.

What the young person may fear	Situations in which an anxiety‐related meaning may go unrecognised
Separation Leaving a vulnerable parent alone (e.g., a parent with mental or physical illness, domestic violence, or marital conflict) Growing up Being bullied at school (Egger et al. 2003) Academic failure (performance anxiety, depression, attention or learning difficulties) Narcissistic injury	Exposure to parental conflict, where absence from school may come to function as a way of bringing the parents together—Psychological suffering that is unrecognised, or that the young person is not able to put into words—An atypical or unclear diagnostic picture, a sense of not fitting in, or a young person whose functioning does not meet the expectations held of them—Bullying already experienced, whether in the past or ongoing

*Note:* Bullying appears in both columns. This is deliberate: anticipating bullying and having been subjected to it call for different clinical responses, the first bearing on what the young person expects of the school, the second on what has already taken place there and on what the institution did or did not do about it.

Attendance problems can also be differentiated by the function the absence serves rather than by its form, a distinction operationalised in the School Refusal Assessment Scale. Reviewing that approach, Heyne et al. (2019) observe that differentiation by function may assist treatment planning while remaining insufficient as a basis for classification. Table 1 proceeds differently, setting out what going to school may come to mean for a young person and what the absence may come to do within family relations. Meaning is what a family can take hold of and elaborate with the clinicians, and it is on that register that the settings described here work.

### 3.2 Parents' Experiences and the Question of “Medical Emergency”

School refusal was traditionally treated as a medical emergency resolved by return to school, a view now regarded as unhelpful. Our clinical experience suggests that many parents of these adolescents themselves report past difficulties with school. The urgency families and schools express is often related to limited familiarity with the psychopathology at work: parents focus on a rapid return rather than on the underlying difficulty, absence disrupts daily life, and schools remind parents of their legal obligation to ensure attendance. This pressure conflicts with therapeutic priorities, since treatment initially requires reducing external pressure so as not to intensify an anxiety that is already paralysing, even in a young person who remains strongly motivated to return.

Reducing this pressure need not be understood as therapeutic delay, and may instead create the conditions for a more sustainable return to school. In our clinical experience, confronting the anxiety trigger too abruptly leads to exhaustion after several weeks or months, and the adolescent internalises repeated failure. The evidence on timing remains thin: reviewing developments since 2000, Elliott and Place (2019) find little substantial advance capable of guiding practitioners. What can be distinguished, however, is a coercive return to school, imposed under pressure and without preparation, from a graded exposure planned with the young person and revised as it proceeds. In our clinical experience, the former may contribute to the exhaustion described above, whereas the latter forms part of a planned therapeutic process.

Reported long‐term outcomes are mixed. In a French clinical account, about one third of adolescents improve partially, one third fully reintegrate into regular schooling, and one third do not return successfully (Benoit et al. 2020). These proportions come from clinical description rather than from a cohort study and should be read as indicative. A substantial minority require longer‐term psychiatric care, the main challenge being the prevention of social phobia (Heyne et al. 2019).

From our local clinical perspective, described in a previous publication by our team (Radjack et al. 2025), it is helpful to explain early that recovery may be slow and that the timing and pace of school reintegration should be adjusted to the adolescent's clinical condition. Intermediate school arrangements may be proposed, or care prioritised according to the recency of the absence, the severity of symptoms and the possibility of a partnership with the school. School exemption is never straightforward: a medical certificate should justify only a temporary absence, and only when accompanied by a care plan.

In France, management of school refusal tends to be highly medicalised. International comparisons suggest that some countries adopt a more educationally centred approach (Heyne and Brouwer‐Borghuis 2022). In our view, medical treatment should not exclude complementary educational support when appropriate.

The most common strategy is a gradual reintegration, which may be formalised in an Individualised Schooling Project (Projet d'Accueil Individualisé), documented by a certificate and sometimes accompanied by a confidential medical note for the school nurse or doctor. It is co‐constructed with the adolescent, the parents and the school, and close knowledge of the school system is needed for the recommendations to be realistic: validating the year in stages, or exempting the young person temporarily from homework, oral questioning, grading or particularly stressful subjects. In the longer term, recognition of psychological disability may facilitate further accommodations. Correspondence courses are a last resort: they often reinforce social avoidance and demand an autonomy that may be unrealistic even for high‐achieving students. Intermediate arrangements—combining distance and in‐school classes, home or hospital teaching, or small‐group classrooms—help stabilise a situation before a more permanent plan is built. In our local clinical experience, full‐time correspondence schooling is generally considered only when other arrangements are not feasible because it may reinforce social avoidance and require a level of autonomy that some adolescents find difficult to sustain (Radjack et al. 2025).

Where there is no acute risk, the more pressing task is often not rapid reintegration but calming the family, giving them information, and avoiding such pitfalls as premature full‐time correspondence courses or unconsidered reorientation.

Calming the sense of emergency a family experiences does not mean delaying intervention irrespective of the adolescent's clinical condition. Priorities are set case by case, through an assessment of acute suicidal or medical risk, functional deterioration, psychiatric comorbidity, the duration and course of the absence, previous bullying, family dynamics, and the adolescent's readiness for a collaborative return. Acute risk requires immediate assessment and intervention. In its absence, reducing pressure may create the conditions in which the adolescent and family can engage in a realistic care plan and a graded return. The relevant distinction is therefore not between acting and waiting, but between an intervention adjusted to the adolescent's clinical state and a premature response driven primarily by the anxiety of the surrounding adults.

### 3.3 Clinical Vignette: Nurse Reception at Initiation of Care

Rachel, a 15‐year‐old girl, attended a walk‐in consultation with her father. She had been unable to attend school for 3 weeks because of morning anxiety attacks. Her parents wondered whether this was adolescent fatigue or a passing whim linked to her preoccupation with appearance, while also fearing something more serious. She lived with her parents and a younger brother, was in seconde (tenth grade) and hoped to study law. Since the start of the school year, she had felt increasingly unwell without a clear external trigger. She reported guilt about eating and often skipped meals, with excessive concern about clothing and appearance, and described hypersomnia with nocturnal awakenings, difficulty concentrating, frequent crying, and a pervasive sadness reminiscent of the previous year. She denied suicidal ideation but acknowledged episodes of self‐harm. Her symptoms had worsened in recent weeks, morning panic attacks preventing her from going to school despite her motivation to attend. The nurse also noted a history of bullying in primary school. Rachel asked explicitly for help. Because she reported self‐harm, suicidal risk was explored in an individual interview: she reported no suicidal plan and had no history of suicide attempt, and she maintained friendships and described her family as supportive, both identified as protective factors. These elements informed the assessment of urgency and the care plan that followed. The nurse recommended further evaluation by a physician while making clear to the family that immediate school reintegration should not be the priority until a tailored plan was established. The account stops here: it shows how a first encounter is conducted and what it settles, not how the situation evolved.

The vignette shows the overlap between anxiety‐related school refusal, depressive symptoms, preoccupation with body image and self‐harm, a combination frequent in adolescence and one that complicates management. It also shows how parental readings range from minimising to catastrophising. Rachel's history of bullying adds a further vulnerability, and the setting in which it occurred remained part of what she was being asked to return to. From a nursing perspective, first‐line assessment is not limited to deciding on referral: the nurse assesses psychiatric and somatic risk, distinguishes the adolescent's request from that of the parents, contains the pressure for an immediate solution, and establishes enough alliance for a collaborative plan to become possible. Staged multidisciplinary responses, rather than immediate reintegration, have been described in the intervention literature (Kearney and Graczyk 2020).

### 3.4 Institutional Approaches

At the Maison des Adolescents, the coordinating child psychiatrist assesses each situation and introduces complementary support at the appropriate time. Families frequently feel their child will miss the chance of recovery unless every therapeutic modality is implemented at once. Yet introducing multiple interventions too early may intensify the pressure to recover quickly, and in our clinical experience premature exposure can leave some adolescents with a renewed sense of failure. Clinicians must also remain attentive to their own countertransference, since the anxiety of parents can be mirrored by professionals and reinforce agitation rather than containment.

Table 2 sets out the interests and the modalities of the therapeutic proposals. Individual psychotherapy may be introduced when the adolescent is willing to explore symptoms, and group psychotherapy can offer a containing setting in which to work on peer relationships. More structured institutional care may include weekly day‐hospital programmes, which help adolescents relearn group participation, rediscover the pleasure of learning through therapeutic mediation such as music or art, and refine a reintegration plan gradually.

**TABLE 2:** Interests and modalities of therapeutic proposals in our institution.

Therapeutic modality	Content	Benefits/aims	Risks/limitations
Weekly Day Hospital	One‐day‐per‐week support program—Transitional space between school and home—Staged objectives—Group exposure (≈8 adolescents)—Educational workshops	Provides a structured third‐party environment—Facilitates emotional expression through cultural mediation—Offers a reference nurse (support for the adolescent and family, liaison with school and healthcare sectors)—Supports gradual reintegration into schooling (educational workshops or hospital‐based classes)	Time‐consuming (not suitable for recent dropouts who may return to school more quickly)—May install the adolescent in institutional life rather than support movement out of it, which is why it suits a prolonged withdrawal better than a recent one—Requires availability for one full day per week—Families may expect rapid re‐schooling, highlighting the need to assess readiness for treatment
Multifamily Therapy (MFT)	Monthly sessions (9 × 3 h)—Includes siblings—Heterogeneous groups—Explores family resources and the role of the problem within family history—Multiple formats (single‐family, sibling subgroups, generational, inter‐family)	Promotes better understanding of the adolescent's suffering behind school refusal—Reduces parental guilt by enabling exchanges with other parents—Reactivates family development and future planning (school, training, care)—Recognises the adolescent's strengths and resources despite dropout—Shifts focus away from the problem within the family—Breaks social isolation, enhances therapeutic alliance—Encourages parental letting‐go and self‐care	Requires the whole family to be available for a half‐day session—Does not address specific family dynamics (better suited to single‐family therapy)—Mobilises a whole family for a difficulty that, in some situations, does not belong to the family—Demands high coordination and experienced therapists
Transcultural Consultation	Group setting bringing together co‐therapists from different professions and cultures—A nurse acts as referring professional, mediator and co‐therapist—Link maintained with the family's other consultations—Family placed in an active position to build meaning and solutions with the therapists	Widens the explanatory frame beyond medical nosography—Holds psychological, cultural and social readings together without ranking them—Restores the family's part in the work and relieves parental guilt—Allows the content of thought to circulate again within the family	May turn a scholastic difficulty into a cultural one when the family had not raised culture—Resource‐intensive and dependent on the availability of several co‐therapists—Requires specialised training and is not widely available
Full‐Time Hospitalisation	Indicated by acute psychiatric or medical risk, marked functional deterioration, or a comorbidity that cannot be assessed or treated on an outpatient basis—Immersion in institutional life—Work on underlying issues—Assessment of comorbidities—Management of severe cases—Containment of separation anxiety—Preparation for transfer to a study‐care center	Allows underlying problems to surface—Facilitates comprehensive clinical assessment—Suitable for severe clinical presentations	Rarely indicated for school refusal alone—Risk of dependency on institutional life—Limited direct impact on school reintegration

*Note:* The four modalities are the resources of a single service. Each is introduced at a particular moment of a care trajectory and on its own indication. For each, the table sets out what it consists of, what it aims at, and what constrains its use.

Multifamily therapy is a further option, bringing together six to eight families and a multidisciplinary team of nurses, psychologists and psychiatrists over nine three‐hour sessions. Each workshop takes up a theme, integrating parents, patients and siblings through role‐play and storytelling and often positioning adolescents as experts. The format can reframe the problem, reduce parental helplessness and foster renewed family dynamics (Snegaroff et al. 2024, 2025; Roué et al. 2021).

Full‐time hospitalisation is occasionally considered. Its indication is not school refusal in itself but acute psychiatric or medical risk, marked functional deterioration, or a comorbidity that cannot be assessed or treated on an outpatient basis. Comprehensive psychological assessment may also help clarify comorbidity, revive a stalled treatment, or reassure an adolescent about social or learning skills (Mazeaud et al. 2023).

Active family involvement is a principle across these modalities. Involving parents reduces guilt and allows them to re‐appropriate a modified parental role (Snegaroff et al. 2024). It is a principle of the settings described here rather than a practice whose effect on outcomes we have evaluated.

## 4 Transcultural Perspectives

### 4.1 Anxiety‐Related School Refusal in Immigrant Adolescents

Our clinical work resonates with observations in the literature that school refusal and absenteeism are categorised differently across social contexts: in large cities and more affluent areas, anxiety‐related school refusal tends to be described as a care‐related problem, whereas in working‐class and migrant‐dense areas absenteeism is more often framed as an educational one (Martin et al. 2020; Rosenthal et al. 2020). Accurate recognition is clinically consequential, since mislabelling anxiety‐related school refusal as truancy may leave psychiatric suffering unattended.

School places cognitive, emotional, and identity demands on adolescents. For some young people in migration contexts, academic achievement may carry particular significance, linked by adolescents or their families to expectations of social mobility and integration in the host country. However, in the process of identity construction, adolescents may find themselves caught between their parents' aspirations for integration, their own emerging autonomy, and the fear of cultural loss. In some families, for reasons that may include the colonial histories shaping the relation between the two languages and the two countries, the culture of the host society is valorised over the culture of origin. Children may then take on mediating functions, translating and negotiating with institutions, that reverse the ordinary distribution of roles between generations. Where this occurs, it can become a source of conflict in adolescence. It is one configuration among several, and migration produces no single pattern of its own.

In a qualitative study conducted in France with adolescents and young adults from immigrant and ethnic minority backgrounds, Brault et al. (2022) identified four themes in the accounts of its participants: perceived failure to meet parental expectations, shame and guilt, social marginalisation through absenteeism, and parental experience of discrimination within schools. Each is a question to put to a family rather than a description of one, and a given consultation may find that none of them applies. They converge, however, on the place of parents in the care itself, both in relieving guilt and in restoring their standing as protective figures.

Attending to cultural meanings does not displace structural determinants, and the two should not be substituted for one another. Families in this situation may also face discrimination in their contacts with school and health institutions, socioeconomic insecurity, unstable housing, and practical barriers to care including appointment systems, waiting times, language, and the cost of travel. Rosenthal et al. (2020) describe parental distress and cultural barriers in access to care among migrant parents of adolescents with school refusal, and Martin et al. (2020) document how school personnel may read the absence of children from immigrant families as truancy rather than as a clinical problem. Misrecognition therefore operates at the level of institutions as much as within families, and a transcultural approach that did not address this would leave the larger part of the problem untouched. The psychic configurations at stake when school investment meets questions of loyalty, filiation and debt are developed at greater length elsewhere (Rizzi et al. 2026).

### 4.2 Clinical Vignette: Lucía in Transcultural Consultation

The transcultural consultation system (Moro 2020) brings together several co‐therapists from different professions and cultures, so that the same situation can be given more than one account—a cultural one and a medical one, for instance—each complete in its own terms rather than completing the other. A nurse is always the referring professional for the transcultural consultation, acting as the link with the family's other consultations, as a mediator, and as a co‐therapist. This system puts families in an active position to build meaning and solutions together with therapists.

Lucía, a 16‐year‐old born in France to Peruvian parents, was referred to a transcultural consultation for recurrent somatic complaints without organic cause. The family participated in about 10 transcultural consultations. Lucía had withdrawn from school for several months, with few social connections, and reported a traumatic assault by a peer. This compounded her isolation. The consultation occurred in the post‐COVID period, which reactivated the family's traumatic memories of curfews and violence during Peru's internal political conflict. Lockdown measures triggered avoidance and confinement at home, making Lucía unable to return to school even after restrictions were lifted, a phenomenon also observed in other adolescents with prior trauma (Li et al. 2024). Her early schooling was marked by her mother's hidden battle with cancer, alongside language barriers and the father's long working hours. Lucía recalled difficulty separating from her exhausted mother, reinforcing school avoidance as a means of staying close to her.

Episodes of loss of consciousness had occurred before the transcultural consultations and had taken Lucía to an emergency department. Electroencephalogram, brain MRI, and neurological assessment identified no neurological cause, and in view of the clinical context, the team retained a dissociative, trauma‐related hypothesis. The episodes became progressively less frequent over the 10 consultations, particularly after Lucía was able to disclose a specific traumatic event.

In some consultations Lucía remained uncomfortable, dissociated, and sleepy. The nurse attended to the nonverbal narrative and adjusted the care at moments of loss of consciousness. What gave new impetus to a situation frozen by successive traumas was the mother's account of the migration, the work on post‐traumatic aspects, the unspoken words of the family and the return to parental experience. Lucía was confronted with a situation of acculturation in which the cultural meanings of the host country met those transmitted within the family. The framework we work from distinguishes filiation, what is transmitted through parental lineage, from affiliation, what is acquired through belonging to social groups, and examines how migration may render the articulation between them particularly visible, plural, or conflictual. Read within that framework, the interruption in this family's account of its own history, which the parents linked to shame and to events in Peru, could be understood as having limited what was available to Lucía for making sense of her situation. Such a reading belongs to the framework that produced it, and in the consultation it is offered to the family alongside others rather than in their place.

This vignette illustrates one way in which anxiety‐related school refusal may become intertwined with processes of acculturation, trauma and family dynamics in a migration context, and one of the sites at which the difficulty of holding together several influences, and of accepting an identity built from more than one source, can become visible. The situations leading to school refusal are diverse, and the clinician holds several diagnostic hypotheses at once. In migration situations the transcultural approach widens the explanatory frame beyond medical nosography, validating psychological, cultural and social readings at once and restoring the family's part in the work. However, such consultations are resource‐intensive, require specialised training and are not widely available.

## 5 Discussion

Across the clinical situations and the therapeutic settings described above, one issue recurs: the level and the intensity of the intervention need to correspond to the level at which the difficulty is operating. Urgency is not synonymous with severity, and a more comprehensive response is not necessarily a better one.

### 5.1 Matching the Level of Intervention to the Clinical Problem

Each of the settings described here carries a risk that mirrors what it is designed to address. Day‐hospital care can install an adolescent in institutional life instead of moving them out of it, which is why it suits a prolonged withdrawal better than a recent one that may resolve more quickly. Multifamily therapy mobilises a whole family for a difficulty that, in some situations, does not belong to the family. And a transcultural consultation can turn a scholastic difficulty into a cultural one when the family had not raised culture at all. Table 2 sets out the corresponding limitations of each setting. A recent withdrawal without acute risk may call for containment, assessment and a negotiated return rather than institutional escalation, where a longstanding withdrawal with severe comorbidity may call for considerably more. The clinical task is therefore less to intervene early than to identify where the difficulty is operating—within the adolescent, within family relationships, within the school environment, or in their interaction—and to calibrate the response to it. Intervening too early, or at a level that does not fit the situation, is as much a clinical error as intervening too late.

### 5.2 Global Perspectives on School Refusal

School refusal occurs worldwide, with prevalence estimated at 1%–2% of school‐aged children and 5%–15% in clinic‐referred samples (Egger et al. 2003). The categories through which non‐attendance is recognised and managed nevertheless vary across psychiatric, educational, and cultural contexts. Comparable descriptions from low‐ and middle‐income countries are scarce, which limits what can be said about the phenomenon outside high‐income settings and underlines the value of a transcultural lens in research as in care.

School refusal is therefore not simply an individual psychiatric issue but a systemic and culturally embedded one, and the Maison des Adolescents, where nurses are the first professionals a family meets, offers one framework for addressing it.

### 5.3 Strengths and Limitations

This paper is grounded in the French context, specifically within a Maison des Adolescents located in a university hospital. The main strength of this contribution lies in its unique clinical experience within an institutional model that emphasises psychodynamic and systemic approaches, while also integrating nursing, medical, and transcultural perspectives.

Several limitations bear on what this account can establish. We are the clinicians of the centre we describe, and what we notice is shaped by the way we have learned to work. A specialised service attracts the situations that suit its way of working, so the configurations presented here are not a representative picture of anxiety‐related school refusal. The resources the centre disposes of—transcultural consultation, multifamily therapy, day‐hospital care, a coordinating child psychiatrist—are unevenly distributed, and how much of the model would survive their absence remains an open question. The terminological difficulty complicates comparison across settings, and the frequent and heterogeneous comorbidities encountered, from depression to somatisation and eating disorders, make homogeneous care pathways difficult to propose. The literature discussed was assembled around the themes of the paper rather than through a systematic search. Finally, the approach holds several readings of a situation alongside one another without ranking them, and this is deliberate, since no register subsumes the others; it follows that we cannot say which reading carries the decisive weight in a given case. The model itself has not been evaluated, unlike a comparable French day‐hospital programme recently assessed in a Phase II trial (Denis et al. 2026).

The theoretical and therapeutic perspectives discussed here were selected purposively, because they derive from the institutional model of the French Maisons des Adolescents and structure the clinical work of our centre. That model is developmental and age‐based rather than organised around a single diagnosis: adolescents' difficulties are approached as dynamic and evolving, at the intersection of individual psychopathology, family relationships, school experience and the wider social and cultural environment (Radjack et al. 2026). Psychodynamic, systemic, institutional and transcultural perspectives were retained because they illuminate complementary dimensions of anxiety‐related school refusal: the meanings attached to attendance and absence, family and institutional responses, and the possible influence of migration, language and experiences of discrimination. The modalities presented are the principal ways in which this framework is implemented when standard outpatient care is insufficient or when greater diagnostic, relational or cultural complexity is present. Their selection reflects the orientation of our centre and does not constitute a systematic comparison of available treatments.

### 5.4 Perspectives

Future approaches should strengthen partnerships between schools, care teams and families while preserving flexible educational pathways. Reintegration is not linear: it requires adaptation to the adolescent's evolving condition and a thorough knowledge of available resources, which may include alternative schooling arrangements for young people who cannot return to a conventional institution, or study‐care centres combining academic courses with therapeutic workshops (Guilbert Calonne et al. 2021). Such arrangements are not endpoints. Their indication depends on the adolescent's clinical condition, the family situation and the capacity for reintegration, and their benefits have to be weighed against the risks of prolonged separation from ordinary school and family life.

## 6 Implications for Nursing Practice

What this implies for nursing practice extends beyond our own setting. The value of a first‐line nursing assessment is not only organisational. At first contact the nurse occupies a position from which relational engagement, physical and mental‐health assessment, risk identification, family containment and coordination of care can take place within a single encounter. It builds the alliance on which any later work depends, offers reassurance at a moment when families are frightened, and addresses the young person as a whole rather than as a set of symptoms. Where access to specialist physicians is delayed, it also allows care to begin at first contact rather than at the end of a waiting list. In France, the development of advanced practice nurses in mental health gives this function a widening professional framework, and the principle holds wherever nurses are the first professionals a family meets.

The assessment covers a defined set of areas. Mood, sleep and eating are explored systematically, while others are developed according to the presenting problem. Nurses examine the history and evolution of the difficulties, school functioning, peer relationships and family dynamics. When school attendance has ceased, previous bullying is systematically explored. Their combined physical and mental‐health expertise helps them identify somatic manifestations that may reflect psychological distress while remaining attentive to the need for medical assessment. For example, when weight loss is reported, nurses explore whether it is associated with eating‐disorder cognitions or may instead be secondary to depression. The interview uses simple, developmentally appropriate language and is not organised as the systematic administration of a standardised questionnaire, in order to preserve its relational quality and adapt the assessment to the adolescent's presentation.

Nurses can also contribute to continuity of care by maintaining communication with adolescents, families and the multidisciplinary team and, where appropriate and with consent, with school professionals. In anxiety‐related school refusal, this coordinating role can support collaborative, graded school reintegration, with steps adjusted to the adolescent's clinical condition and reviewed over time.

## 7 Conclusion: Individual, Group, and Systemic Approaches

The management of anxiety‐related school refusal must be conceived at three interconnected levels: individual, family and environmental. This is what makes prevention, early detection and systemic care important. School refusal should not be reduced to a psychiatric symptom alone; it also reflects broader tensions within families, schools and society.

Addressing it requires an integrated vision combining psychological, educational, and social dimensions. Nurses carry much of this work: as first‐line caregivers they contain parental urgency, guide families through uncertainty, and mediate between school and healthcare teams, building the trust on which a workable care plan depends. This paper has described the clinical experience and the reasoning of one centre; the principles it emphasises, graded intervention and attention to the level at which a difficulty is operating, are offered for discussion, and establishing their transferability would require comparative work that remains to be done.

## Author Contributions

**Rahmeth Radjack:** conceptualization, writing – original draft, writing – review and editing, supervision. **Alice Titia Rizzi:** writing – review and editing. **Nelly Massari:** investigation, writing – original draft. **Delphine Brown:** writing – review and editing. **Aurélie Harf:** writing – review and editing. **Eglantine Mazeaud:** writing – review and editing. **Christine Baveux:** resources. **Marie Rose Moro:** supervision, writing – review and editing.

## Funding

This article received no specific grant from any funding agency in the public, commercial, or not‐for‐profit sectors. The clinical research programme within which the transcultural situation falls was sponsored by Assistance Publique—Hôpitaux de Paris and funded by the Programme Hospitalier de Recherche Clinique 2018 of the French Ministry of Health.

## Ethics Statement

The two situations do not have the same research status. The transcultural situation (Lucía) forms part of the EDPT‐ADOS clinical research programme evaluating transcultural psychotherapy, sponsored by Assistance Publique—Hôpitaux de Paris and funded by the Programme Hospitalier de Recherche Clinique 2018 of the French Ministry of Health (ClinicalTrials.gov NCT04206969), approved by the Comité de Protection des Personnes Sud Méditerranée IV on 13 January 2020 under reference CPP 19.10.01. For that situation, the adolescent and her parents gave written informed consent to the publication of anonymised material. The first‐contact nursing situation (Rachel) does not form part of a research protocol. It is a de‐identified clinical account drawn from routine care.

## Consent

This article is a perspective article and reports no original empirical data. The two clinical situations are individual situations, not composite or amalgamated accounts; each was written by the clinician responsible for that young person, and both clinicians are co‐authors of this article. Both adolescents agreed to the publication of their respective clinical vignettes, and consent was obtained from their parents. In each case this consent covers the publication of de‐identified clinical material. Both accounts have been de‐identified. Rachel and Lucía are pseudonyms, and identifying details have been altered wherever the alteration does not affect the clinical point being made. No photograph or other potentially identifiable material is reproduced, and neither young person could be recognised from the details given.

## Conflicts of Interest

The authors declare no conflicts of interest.

## Data Availability

The data that support the findings of this study are available on request from the corresponding author. The data are not publicly available due to privacy or ethical restrictions.
